# Actin Alpha 2, Smooth Muscle (ACTA2) Is Involved in the Migratory Potential of Malignant Gliomas, and Its Increased Expression at Recurrence Is a Significant Adverse Prognostic Factor

**DOI:** 10.3390/brainsci13101477

**Published:** 2023-10-19

**Authors:** Takumi Hoshimaru, Naosuke Nonoguchi, Takuya Kosaka, Motomasa Furuse, Shinji Kawabata, Ryokichi Yagi, Yoshitaka Kurisu, Hideki Kashiwagi, Masahiro Kameda, Toshihiro Takami, Yuko Kataoka-Sasaki, Masanori Sasaki, Osamu Honmou, Ryo Hiramatsu, Masahiko Wanibuchi

**Affiliations:** 1 Department of Neurosurgery, Osaka Medical and Pharmaceutical University, Osaka 569-8686, Japan; 2 Department of Pathology, Osaka Medical and Pharmaceutical University, Osaka 569-8686, Japan; 3 Department of Neural Regenerative Medicine, Research Institute for Frontier Medicine, Sapporo Medical University School of Medicine, Hokkaido 060-8556, Japan

**Keywords:** high-grade glioma, actin alpha 2, smooth muscle (ACTA2), actin alpha, cardiac muscle 1 (ACTC1), cytoskeleton, cell migration, recurrence

## Abstract

Malignant glioma is a highly invasive tumor, and elucidating the glioma invasion mechanism is essential for developing novel therapies. We aimed to highlight actin alpha 2, smooth muscle (ACTA2) as potential biomarkers of brain invasion and distant recurrence in malignant gliomas. Using the human malignant glioma cell line, U251MG, we generated ACTA2 knockdown (KD) cells treated with small interfering RNA, and the cell motility and proliferation of the ACTA2 KD group were analyzed. Furthermore, tumor samples from 12 glioma patients who underwent reoperation at the time of tumor recurrence were utilized to measure ACTA2 expression in the tumors before and after recurrence. Thereafter, we examined how ACTA2 expression correlates with the time to tumor recurrence and the mode of recurrence. The results showed that the ACTA2 KD group demonstrated a decline in the mean motion distance and proliferative capacity compared to the control group. In the clinical glioma samples, ACTA2 expression was remarkably increased in recurrent samples compared to the primary samples from the same patients, and the higher the change in ACTCA2 expression from the start to relapse, the shorter the progression-free survival. In conclusion, ACTA2 may be involved in distant recurrence in clinical gliomas.

## 1. Introduction

Malignant gliomas are characterized by aggressive tumor cell proliferation and a poor prognosis despite treatments, such as surgical resection, radiotherapy, and chemotherapy, with reported 5-year survival rates of approximately 4.7–10.1% for World Health Organization (WHO) grade 4 glioblastomas (GBM) [1,2]. Patients with malignant gliomas present with frequent recurrences not only locally but also in distant areas of the brain [3]. One of the factors limiting complete surgical resection and causing poor prognosis is attributed to glioma cell infiltration. Many studies have reported on the invasion of malignant gliomas; for example, malignant glioma cells modify the extracellular matrix (ECM) by releasing matrix metalloproteinases (MMPs) and coding and non-coding RNAs via extracellular vesicles, thereby remodeling it into an environment more stable for invasion [4,5,6]. Elucidating the mechanisms controlling the invasion and recurrence of malignant gliomas holds great promise for improving prognosis.

In this study, we focused on actin family genes as potential biomarkers of brain invasion and distant recurrence of gliomas. The major components of the cytoskeleton are actin filaments, which are also essential functional proteins that form the molecular apparatus responsible for cell motility and intracellular material transport [7]. In terms of glioma cell invasion described in the previous example, malignant glioma cells need to modify their contacts with the ECM, which involves the actin cytoskeleton [6]. Six isoforms of actin derived from six paralog genes exist, and numerous associations have been reported between actin isoform expression in varying cancers and prognosis or resistance to cancer drug treatment [8]. One of these isoforms, high expression of actin alpha, cardiac muscle 1 (ACTC1), is a significant poor prognostic factor in malignant gliomas [9]. We previously reported that ACTC1 knockdown (KD) in a human malignant glioma cell line inhibited cell migration ability [10].

Thus, we hypothesize that actin isoforms other than ACTC1 also involve cell migration and recurrence in malignant gliomas. Actin alpha 2, smooth muscle (ACTA2), one of the actin isoforms, is mainly found in the smooth muscle of the vascular system and is involved in its contractility [11]. This study aimed to investigate the role of ACTA2 in cell migration of glioma cells and determine the impact of ACTA2 expression level on prognosis and recurrence in glioma patients.

## 2. Materials and Methods

### 2.1. Cell Culture

U251MG, a human GBM cell line, was purchased from the Japan Collection of Research Bioresources Cell Bank (National Institutes of Biomedical Innovation, Health, and Nutrition, Osaka, Japan). The cells were cultured in Dulbecco’s Modified Eagle’s Medium supplemented with 10% fetal bovine serum, penicillin, streptomycin, and amphotericin B at 37 °C in an atmosphere of 5% CO_2_.

### 2.2. Droplet Digital Polymerase Chain Reaction (dd-PCR)

According to the manufacturer’s protocol, total RNA was extracted using the RNeasy Mini Kit (Qiagen, Hilden, Germany). A NanoDrop Lite spectrophotometer (Thermo Fisher Scientific Inc., Waltham, MA, USA) was used to measure the total RNA concentration and A260/A280 ratio. Samples with A260/A280 ratios < 1.8 were excluded. The QuantiTect Reverse Transcription Kit (Qiagen) was used for cDNA synthesis via RNA reverse transcription. A water/oil emulsion droplet in combination with a microfluidic device was used to conduct the dd-PCR analysis. To measure the ACTC1 expression levels, PCR mixtures with a final volume of 20 μL were prepared using 8 μL DNA (dilution: 2:6), 10 μL Digital PCR Supermix for Probes (Bio-Rad, Hercules, CA, USA), and 1 μL Bio-Rad Prime PCR primer assays for ribosomal protein L37 (RPL37) (dHsaCPE5037980; Bio-Rad) and ACTC1 (dHsaCPE5049966; Bio-Rad). To measure ACTA2 expression levels, PCR mixtures of 20 μL final volume were prepared using 8 μL DNA (dilution: 1:7), 10 μL Digital PCR Supermix for Probes (Bio-Rad), and 1 μL PCR primer assays for ribosomal protein L37 (RPL37) (dHsaCPE5037980; Bio-Rad) and ACTA2 (dHsaCPE5051320; Bio-Rad). Each droplet was amplified using PCR (C1000 Touch Thermal Cycler; Bio-Rad) after each sample was divided using a droplet generator. The thermal cycling conditions were the following: 95 °C for 10 min, 39 cycles of extension at 95 °C for 30 s/cycle, and 57 °C for 1 min, followed by 98 °C for 10 min. The target gene concentration was assessed by loading a 96-well PCR plate onto a QX200 droplet reader (Bio-Rad) after amplification. PCR data were analyzed to measure the number of droplets positive or negative for the ACTC1, ACTA2, and RPL37 probe in each sample using QuantaSoft (version 1.7.4, 2014) (Bio-Rad) analysis software. Using a Poisson algorithm to determine the target concentration, we calculated the proportion of target positive droplets. RPL37 was used as the reference gene for quantitative evaluation, and the results were expressed as the ratio of ACTC1 to RPL37 or ACTA2 to RPL37.

### 2.3. Small Interfering RNA Transfection

Lipofectamine™ RNAiMAX (Thermo Fisher Scientific Inc.) transfection reagent was utilized to transfect ACTC1-specific siRNA (sc-105181; Santa Cruz Biotechnology, Dallas, TX, USA) or/and ACTA2-specific siRNA (sc-43590; Santa Cruz Biotechnology) into U251MG following the manufacturer’s protocol. ACTC1 knockdown alone (ACTC1-KD) cells were transfected using 3 μL ACTC1-specific siRNA, ACTA2 knockdown alone (ACTA2-KD) cells were transfected using 3 μL ACTA2-specific siRNA, and simultaneous knockdown of ACTC1 and ACTA2 (ACTC1/ACTA2-KD) cells were transfected using 3 μL each of ACTC1- and ACTA2-specific siRNA, respectively. As a negative control (NC), equal amounts of scrambled siRNA (sc-37007; Santa Cruz Biotechnology) and Lipofectamine™ RNAiMAX transfection reagent were used.

### 2.4. Proliferation Assay

At 24 h after completion of the siRNA transfection protocol, cells were detached and seeded in 6-well plates at 1.0 × 10^5^ cells per well. The number of cells was measured using Countess^®^ II FL (Thermo Fisher Scientific Inc.) after the cells were incubated at 37 °C in a 5% CO_2_ atmosphere for 72 h. The doubling time for each cell was calculated through the measurement of the cell count after 72 h of incubation.

### 2.5. Migration Assay

Cells were seeded into 6-well plates at a density of 5.0 × 10^4^ cells per well 24 h after the knockdown process. Following an additional 24 h incubation period, time-lapse imaging was conducted every 10 min for 18 h while cells were incubated at 37 °C in a 5% CO_2_ atmosphere. For each group, eight regions of interest (ROIs) were defined, and the movement of cells within each ROI was analyzed using the Live Cell Imaging System SI8000 (Sony, Tokyo, Japan).

### 2.6. Immunohistochemistry

U251MG cells were immunostained using an ACTC1 antibody (GTX101876; GeneTex, Inc., Irvine, CA, USA) at a dilution of 1:500 and ACTA2 monoclonal antibody (1A4, eBioscience™; Thermo Fisher Scientific Inc.) at a dilution of 1:500, following the manufacturer’s protocol. After three washes in phosphate-buffered saline (PBS), sections were incubated using the following secondary antibodies: goat anti-mouse IgG (H + L); Alexa Fluor 488-conjugated; A-11001 (Thermo Fisher Scientific Inc.) diluted at 1:200 and goat anti-rabbit IgG (H + L); Alexa Fluor 546-conjugated; and A-11010 (Thermo Fisher Scientific Inc.) diluted at 1:200. Incubation with secondary antibodies was performed for 2 h at 37 °C in a 5% CO_2_ atmosphere. Subsequently, sections were washed three times with PBS, and the nuclei were stained with ibidi mounting medium with 4′,6-diamidino-2-phenylindole (DAPI) (ib50011; NIPPON Genetics Co, Ltd. Tokyo, Japan). Finally, using a confocal laser microscope (STELLARIS 8; Leica Microsystems GmbH, Wetzlar, Germany), the sections were mounted on coverslips and observed.

### 2.7. Clinical Tumor Samples and Medical Information

A total of 50 samples were acquired from consecutive patients who underwent a brain tumor resection at our hospital between 2014 and 2017 and were initially diagnosed with WHO grade 3 or 4 gliomas. Moreover, 24 samples were collected from 12 patients who underwent surgery at our hospital between 2014 and 2022. These patients were initially diagnosed with WHO grade 3 or 4 gliomas, manifested tumor recurrence, and subsequently underwent tumor resection because of relapse at our hospital during the same periods. All samples were cryopreserved at −80 °C in an ultra-low-temperature freezer immediately after resection. Using the method described earlier (Section 2.2), we determined the ACTA2 expression levels in the cryopreserved samples. Clinical information of these patients, such as age, sex, pathology, progression-free survival (PFS), overall survival (OS), characteristics of initial magnetic resonance imaging (MRI) findings, and postoperative therapy information, was extracted from their medical records. The institutional ethics committee approved this retrospective observational study (Osaka Medical and Pharmaceutical University 2022-189). An opt-out method was used to obtain patient consent for this study because of the retrospective nature of this study and the use of anonymized clinical data. Written informed consent for treatment was obtained from each patient.

### 2.8. Validation Using Data from the TCGA Database’s Cohort of Glioma Patients

To investigate the correlation between ACTA2 expression levels and the prognosis of recurrent gliomas, we analyzed an independent patient cohort from the TCGA Research Network (https://www.cancer.gov/tcga: accessed on 31 August 2023), distinct from our dataset. The data were retrieved and analyzed through the GlioVis database (http://gliovis.bioinfo.cnio.es: accessed on 31 August 2023) [12]. First, “TCGA_GBMLGG”, a WHO grade 2–4 clinical glioma dataset consisting of 667 samples, was used. All patients were adults, and samples were not differentiated by histology, tumor subtype, gender, and IDH status. Next, “TCGA_GBM” was used as a dataset confined to WHO grade 4, which contained 357 and 12 samples of primary and recurrent GBM, respectively. Samples used for analysis were restricted to IDH-wildtype and were not differentiated by tumor subtype or gender. HG-U113A was selected as the platform for gene expression.

We evaluated the impact of ACTA2 expression on the overall survival (OS) of glioma patients by categorizing them into two groups: ACTA2 high and ACTA2 low expression groups. Maximum selection rank statistics were employed to determine the optimal cut-off values of ACTA2 expression for these groupings.

### 2.9. Statistical Analysis

The data were subjected to statistical analysis and reported as mean values ± standard deviation or median with range. All statistical analyses were performed using JMP Pro (version 16.2.0) software (SAS Institute Inc., Cary, NC, USA). Statistical significance was determined at a threshold of *p* < 0.05.

## 3. Results

### 3.1. ACTC1 and ACTA2 KD through siRNA Transfection

**Figure 1:**
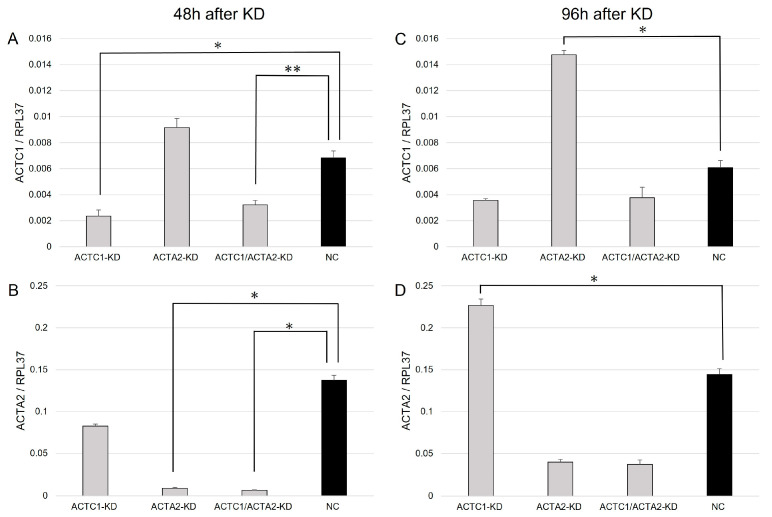
Droplet digital PCR analysis of actin alpha, cardiac muscle 1 (ACTC1) and actin alpha 2, smooth muscle (ACTA2) expression in U251MG cells. (**A**) ACTC1 siRNA-treated cells (ACTC1-KD and ACTC1/ACTA2-KD) demonstrated a significant decline in ACTC1 gene expression compared to negative control (NC) cells at 48 h after transfection. (**B**) ACTA2 siRNA-treated cells (ACTA2-KD and ACTC1/ACTA2-KD) revealed a significant decrease in ACTA2 gene expression compared to NC cells at 48 h after transfection. (**C**) ACTA2-KD cells revealed an increased ACTC1 gene expression compared to NC cells at 96 h after transfection. (**D**) ACTC1-KD cells showed increased ACTA2 gene expression compared to NC cells at 96 h after transfection. *, *p* < 0.001. **, *p* = 0.001. The Student’s *t*-test was used to determine the *p*-values.

The dd-PCR analysis demonstrated that the cells treated with ACTC1 and/or ACTA2 siRNA exhibited a decreased expression of each respective gene compared to the NC cells at 48 h post-transfection (Figure 1A,B, *p* ≤ 0.001), which confirmed the successful KD of each gene via siRNA transfection. Additionally, ACTA2-KD cells showed increased ACTC1 expression compared to NC cells at 96 h post-transfection (Figure 1C, *p* < 0.001). Conversely, ACTC1-KD cells exhibited an increased ACTA2 expression at 96 h post-transfection (Figure 1D, *p* < 0.001).

### 3.2. Cell Proliferation of U251MG

**Figure 2:**
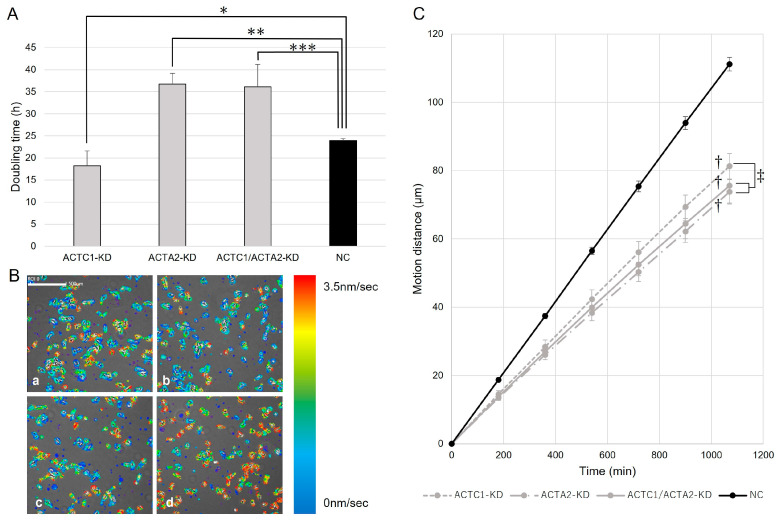
(**A**) The doubling time of each group was calculated. Among the NC and KD cells, the ACTC1-KD cells showed a shorter doubling time (*, *p* = 0.027). Conversely, the ACTA2-KD and ACTC1/ACTA2-KD cells revealed longer doubling times (**, *p* < 0.001, ***, *p* = 0.006). The Student’s t-test was used to perform the statistical analysis. (**B**) The cell motions of each cell were analyzed using time-lapse images acquired using the Live Cell Imaging System SI8000. Areas where movement was detected are color-coded based on movement speed. A visual comparison of knockdown cells (a: ACTC1-KD; b: ACTA2-KD; c: ACTC1/ACTA2-KD) with the NC cells (d) indicated a reduction in the speed of movement in the knockdown cells. Scar bar = 500 μm. (**C**) Significant decreases in KD cells compared to NC cells were noted when calculating the motion distance during the observation period based on the motion velocity (†, *p* < 0.001, Student’s *t*-test). The ACTC1-KD cells demonstrated a significantly longer motion distance than the other knockdown cells (‡, *p* = 0.009, analysis of variance). No significant differences were found between ACTA2-KD and ACTC1/ACTA2-KD cells (Student’s *t*-test).

ACTC1-KD cells showed a shorter doubling time than NC cells (*p* = 0.027), and ACTA2-KD and ACTC1/ACTA2-KD cells revealed longer doubling times compared to that of NC cells (Figure 2A, *p* < 0.001, *p* = 0.006, respectively). No significant difference was found in doubling times between ACTA2-KD and ACTC1/ACTA2-KD cells.

### 3.3. Cell Migration of U251MG

A decrease in cell motion velocity was observed compared to NC cells in ACTC1-KD cells, ACTA2-KD cells, and ACTC1/ACTA2-KD cells (Figure 2B). Similarly, compared to the NC cells, a remarkable reduction in motion distance was noted in the KD cells (*p* < 0.001). When comparing the motion distances between the KD groups, ACTC1-KD cells showed a significantly longer motion distance (*p* = 0.009). However, no significant difference was found in motion distances between ACTA2-KD cells and ACTC1/ACTA2-KD cells (Figure 2C).

### 3.4. Immunohistochemistry

**Figure 3:**
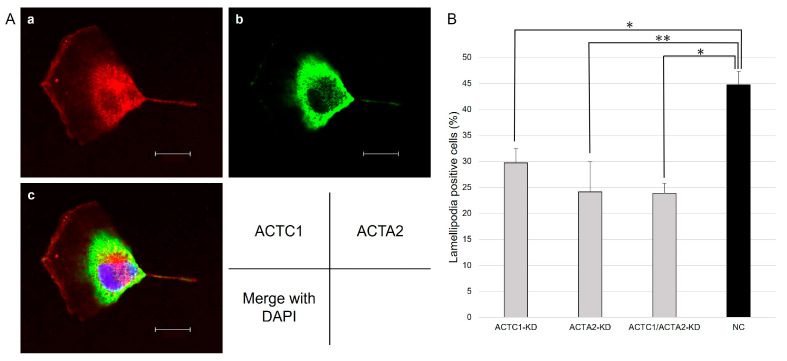
(**A**) Immunocytochemical staining of ACTC1 and ACTA2 in U251MG was visualized via a confocal laser microscope. ACTC1 was noted to accumulate at the lamellipodia, while ACTA2 accumulation at the lamellipodia was less prominent compared to ACTC1 (a: ACTC1 immunostaining; b: ACTA2 immunostaining; c: overlay with DAPI [blue], 600× magnification each). Scar bar = 20 μm (**B**) The rates of lamellipodia formation in each cell were calculated based on the bright-field images obtained simultaneously. ACTC1-KD, ACTA2-KD, and ACTC1/ACTA2-KD cells revealed a reduced lamellipodia formation compared to NC cells. *, *p* < 0.001. **, *p* = 0.001. Statistical analysis was conducted using the Student’s *t*-test to determine the *p*-value.

Lamellipodia are cell membrane protrusions at the leading edge of a cell that extend and migrate parallel to the substrate and are composed of a network of actin filaments [13]. In our study, we noted that ACTC1 exhibited accumulation specifically at the tips of the fan-shaped lamellipodia, while the ACTA2 accumulation in lamellipodia was less pronounced compared to ACTC1 (Figure 3A). The distinct differences in ACTC1 and ACTA2 localization within lamellipodia in U251MG cells are confirmed by these findings.

### 3.5. Lamellipodia Formation of U251MG

The proportion of cells showing lamellipodia formation, as observed in the simultaneously captured bright-field images, was compared among the KD cells and the NC cells. The results indicated that ACTC1-KD, ACTA2-KD, and ACTC1/ACTA2-KD cells revealed a reduced lamellipodia formation occurrence compared to NC cells (Figure 3B, *p* ≤ 0.001).

### 3.6. Clinical Data of Malignant Glioma Patients

The median age at diagnosis was 64.5 years, ranging from 21 to 89 years, among 50 patients with primary malignant glioma. Of these patients, 64% were male. Among the cases, 11 were classified as WHO grade 3, while 39 were stratified as grade 4 gliomas. The median PFS was 9 months, with a range of 1 to 104 months. The median OS was 19 months, also ranging from 1 to 104 months. At the initial presentation, approximately 16% of the patients showed remotely enhanced lesions on Gd contrast-enhanced MRI that were not connected to the fluid-attenuated inversion recovery hyperintense lesions surrounding the main lesion.

Among the 12 patients diagnosed with recurrent malignant glioma, the median age at diagnosis was 48 years, ranging from 30 to 80 years. Of these patients, 75% were male. Among the cases, five were WHO grade 3, while seven were grade 4 gliomas. The median PFS was 8 months, with a range of 4 to 31 months. The median OS was 31.5 months, ranging from 11 to 80 months.

In 11 out of 12 patients, concurrent chemoradiotherapy with 60 Gy of X-ray radiation therapy together with temozolomide was initiated between the initial operation and reoperation, while one patient only received chemotherapy using temozolomide. Malignant glioma patient characteristics are summarized in Table 1. 

### 3.7. ACTA2 Expression in Primary Malignant Glioma

**Figure 4:**
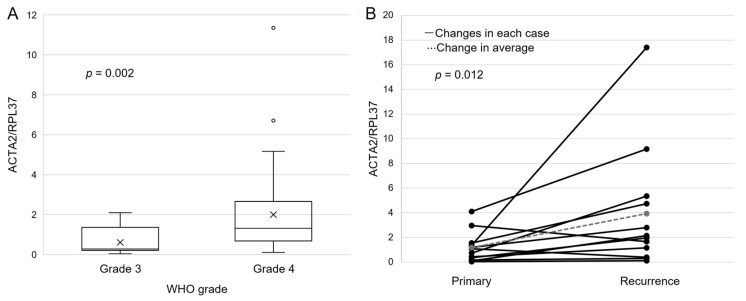
(**A**): ACTA2 expression was assessed in patients with grade 3 primary glioma (*n* = 11) and grade 4 (*n* = 39) patients. ACTA2/RPL37 ratio was significantly higher in grade 4 gliomas. The *p*-values were determined using the Wilcoxon signed-rank test. (**B**): ACTA2 expression changes from the initial diagnosis to recurrence in malignant glioma patients (*n* = 12) were measured. The ACTA2/RPL37 ratio was significantly higher in recurrent malignant glioma than in primary malignant glioma. Moreover, 10 out of 12 cases revealed increased ACTA2 expression during re-currence. Using paired-samples Wilcoxon signed-rank test, *p*-values were determined.

A significant difference was noted when comparing ACTA2 expression levels between WHO grade 3 and grade 4 gliomas: the ACTA2/RPL37 ratio was approximately fourfold higher in grade 4 gliomas, with a median of 1.31 (range: 0.11–11.35) compared to a median of 0.28 (range: 0.04–2.09) in grade 3 gliomas (Figure 4A, *p* = 0.002). Moreover, the ACTA2 high expression group (*n* = 16) revealed a significantly higher proportion of distant lesions, accounting for 31.3% of the brain MRI findings at the initial visit, compared to 8.8% in the ACTA2 low expression group (*n* = 34) (Figure S1, *p* = 0.044).

In the validation cohort study using the TCGA database, WHO grade 4 gliomas (*n* = 153) had significantly higher ACTA2 gene expression than lower-grade gliomas of WHO grades 2 (*n* = 226) and 3 gliomas (*n* = 244) (Figure S2, *p* < 0.001).

When these glioma patients (grades 2–4: *n* = 667) were divided into two groups by ACTA2 expression, those with high ACTA2 expression (*n* = 285) had a significantly worse prognosis than those with low expression (*n* = 382) (Figure S3A, *p* < 0.01). In primary IDH-wildtype GBM, the ACTA2 high expression group (*n* = 138) also had a worse prognosis than the low expression group (*n* = 219) (Figure S3B, *p* = 0.008). 

### 3.8. ACTA2 Expression in Recurrent Malignant Glioma

**Figure 5:**
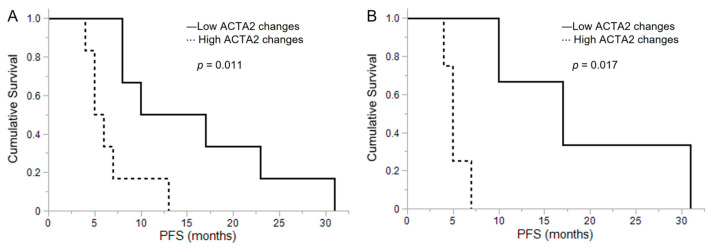
(**A**) The Kaplan–Meier method was used to compare progression-free survival (PFS) between the high and low ACTA2 changes from initial malignant glioma to recurrence. In malignant glioma patients (*n* = 12), the PFS in the high ACTA2 expression change group (*n* = 6) was remarkably shorter than the PFS in the low ACTA2 expression change group (*n* = 6). (**B**) In grade 4 glioma patients (*n* = 7), the PFS in the high ACTA2 expression change group (*n* = 4) was significantly shorter than the PFS in the low ACTA2 expression change group (*n* = 3). Statistical analysis was conducted using the log–rank test to determine the *p*-values.

Comparing ACTA2 expression in initial and recurrent samples from the same glioma patients, we noted that ACTA2/RPL37 increased nearly twofold in recurrent cases (median value: 2.05, range: 0.12–17.39) compared to initial cases (median value: 0.90, range: 0.04–4.10). Specifically, 10 out of 12 patients showed an increased ACTA2 expression from the primary tumors to their corresponding relapsed tumors (Figure 4B, *p* = 0.012). When the PFS from initial disease to relapse was investigated, it was found that the high ACTA2 expression change group (*n* = 6) showed a median PFS of 5.5 months (range: 4–13), which was significantly shorter than a median of 13.5 months (range: 8–31) in the low ACTA2 expression change group (*n* = 6) (Figure 5A, *p* = 0.011). Similarly, when restricting the analysis to grade 4 gliomas, a significantly shorter PFS was noted in the group with a higher change in ACTA2 expression (high change group (*n* = 4): median 5 months (range: 4–7); low change group (*n* = 3): median 17 months (range: 10–31)) (Figure 5B, *p* = 0.017).

In IDH-wildtype GBM in the TCGA database, the group with high expression of ACTA2 at the time of recurrence (*n* = 7) had a significantly worse prognosis than the group with low expression (*n* = 5) (Figure S3C, *p* = 0.001).

## 4. Discussion

In this study, KD of either ACTC1 or ACTA2 in the U251MG glioma cell line led to shorter cell motion distance. Mesenchymal migration, induced by lamellipodia formation, is a type of cancer cell migration [13,14], and we verified that either ACTC1 or ACTA2 KD significantly reduced lamellipodia formation. Previously, we reported that ACTC1 KD in a malignant glioma cell line reduced cell migration [10], and the study results suggest that ACTA2 is also involved in glioma cell migration. ACTA2 was highly expressed in grade 4 gliomas compared to grade 3 gliomas in clinical malignant glioma specimens, and multiple distant brain lesions were more common in the ACTA2 high expression group. These results suggest that ACTA2 may be a therapeutic target in controlling glioma migration and distant recurrence.

ATCA2 has been reported to be a migration-related factor in other cancers and nervous system cells. Furthermore, ACTA2 expression is associated with distant metastasis and poor prognosis in human epidermal growth factor receptor-positive breast, bladder, and colorectal cancers [15,16,17]. Lee et al. elucidated that a significant positive correlation was found between brain metastasis and ACTA2 gene amplification in lung adenocarcinoma and that a high ACTA2 expression is a poor prognostic factor [18]. They also revealed that ACTA2 is involved in metastasis via migration and invasion in lung adenocarcinoma from in vitro and in vivo assays and that ACTA2 silencing suppresses the EMT-related gene, FAK, and c-MET expression [19]. Zhang et al. also reported that ACTA2 downregulation in neural stem cells reduced cell migration [20].

The study revealed that ACTA2 expression was increased in most recurrent cases and that the higher the increase, the shorter the PFS. These results suggest that ACTA2 is involved in malignant glioma recurrence. Several hypotheses were generated regarding the underlying mechanism by which ACTC2 is involved in malignant glioma recurrence.

Malignant gliomas present with a poor prognosis, which is attributed in part to deep brain invasion, limiting complete surgical resection. As mentioned above, one of the migration factors in malignant gliomas is ACTA2, and one hypothesis for the involvement of ACTA2 in recurrence is that cells showing a higher ACTA2 expression may migrate and invade deeper into the brain and escape surgical resection.

Additionally, most of the patients in this study received a combination of anti-cancer drugs (temozolomide) and radiotherapy, referred to as the Stupp regimen, between the initial surgery and reoperation [21]. Another hypothesis is that ACTA2 is involved in resistance to the Stupp regimen. A number of reports exist elucidating actin and resistance to anti-cancer drugs: Che et al. reported that a high ACTC1 expression is associated with low sensitivity to the mitogenic inhibitor paclitaxel in non-small cell lung cancer [22], and Yang et al. reported that ACTC1 is a hub gene conferring chemotherapy resistance to a variety of tumors and its expression is upregulated in multidrug-resistant breast cancer cells [23]. Although one of the standard postoperative treatments for malignant gliomas is the Stupp regimen, which has been shown to prolong PFS and OS, malignant gliomas have also been reported to have a poor prognosis even after receiving the Stupp regimen [24]. Thus, understanding the mechanisms of resistance to radio-chemotherapy in malignant gliomas is crucial. 

The present study shows that ACTA2 expression is a factor in the mechanism of malignant glioma recurrence and represents a challenge for future elucidation of the mechanism of malignant glioma recurrence and control.

In this study, we initially generated U251MG glioma cells in which one of the two α-actin paralog genes, ACTC1 and ACTA2, was knocked down. Interestingly, KD of either α-actin resulted in a remarkable upregulation of the expression of the other α-actin gene. Accordingly, ACTC1-KD cells demonstrated a suppressed ACTC1 expression and enhanced ACTA2 expression compared to control cells, while ACTA2-KD cells demonstrated a suppressed ACTA2 expression and enhanced ACTC1 expression. This fact implies that the ACTC1 and ACTA2 gene expression is controlled by a complementary expression regulation system, suggesting that there may be some biologically purposive reason for compensating for the decline in one α-actin through the increased expression of the other. We added U251MG glioma cells with a simultaneous KD of the ACTC1 and ACTA2 genes as “ACTC1/ACTA2-KD cells” in vitro assays to explore the significance of this complementary regulation of ACTC1 and ACTA2 expression.

The evaluation results of ACTC1 gene expression, ACTA2 gene expression, cell proliferation ability, cell migration capacity, and lamellipodia formation ability in the three KD cell lines compared to control cells (sham-KD) are summarized in Table 2.

In evaluating cellular proliferative capacity, ACTC1-KD cells demonstrated an increased proliferative capacity, while ACTA2-KD and double KD cells showed a significantly decreased proliferative capacity. Among these three types of KD cells, only ACTC1-KD cells with a significantly increased expression of ACTA2 compared to control cells revealed increased cell proliferation, while ACTA2-KD and ACTC1/ACTA2-KD cells with a suppressed ACTA2 expression demonstrated a significantly decreased cell proliferation. These results suggest that ACTA2 has a stimulatory effect on glioma cell proliferation, while ACTC1 expression has a minimal effect on cell proliferation.

It has been reported that ACTC1 is not involved in cell proliferation in malignant glioma [9]. The acquisition of ACTA2 expression has been reported in activated cancer-associated fibroblasts in oral and pancreatic cancer, which are known to be involved in cancer cell proliferation [25,26,27], and it is conceivable that ACTA2 is also involved in cell proliferation in malignant glioma through a similar mechanism. 

Knockdown of either ACTC1 or ACTA2 also inhibited lamellipodia formation, but immunocytochemistry revealed differences in the distribution of ACTC1 and ACTA2 in lamellipodia. This result suggests that each may play an independent role in lamellipodia formation. Taken together, our data suggest that the regulation of ACTC1 and ACTA2 expression is complementary but that their biological roles are not identical.

Six actin isoforms (paralog genes) exist, and the tissues in which they are significantly expressed vary by isoform [8,28]. The interaction between expression and function among actin isoforms has already been reported in several cases: actin gamma 2, smooth muscle (ACTG2) is overexpressed in ACTC1 knockout mice [29]. Moreover, it has also been reported that transgenic ACTC1 expression in actin alpha skeletal muscle (ACTA1) knockout mice restores lethality and muscle weakness associated with ACTA1 loss [30]. Elucidating the mechanisms of interaction between actin isoforms will provide a springboard to explore the mechanisms of cancer migration and proliferation, including in malignant gliomas.

This study has limitations that include the retrospective nature of the study. Another limitation is the limited number of cases where initial and recurrent samples were available from the same patient.

## 5. Conclusions

The present study revealed that ACTA2 is an important migratory factor in malignant gliomas and is involved in recurrence. Elucidation of the migration mechanism in malignant gliomas is crucial in developing future therapeutic regimens, and ACTA2 is a promising candidate as a therapeutic target. Additionally, the interaction between actin isoforms in cancer can be confirmed via the present study and previous reports. Exploring the mechanisms of interaction between actin isoforms may provide a clue to understanding the migration and proliferation mechanisms of cancers, including malignant gliomas.

## Figures and Tables

**Table 1:** Malignant glioma patient characteristics.

		Primary Malignant Glioma (*n* = 50)	Recurrent Malignant Glioma (*n* = 12)
Age at diagnosis, median (range)		64.5 (19–91)	48 (30–80)
Sex			
	Male	32 (64%)	9 (75%)
	Female	18 (36%)	3 (25%)
WHO grade			
	3	11 (22%)	5 (42%)
	4	39 (78%)	7 (58%)
PFS (month), median (range)		9 (1–104)	8 (4–31)
OS (month), median (range)		19 (1–104)	31.5 (11–80)
Postoperative therapy			
	Radiotherapy	48 (96%)	11 (92%)
	Chemotherapy	48 (96%)	12 (100%)

**Table 2:** Summary of in vitro study.

	ACTC1 Expression	ACTA2 Expression	Cell Proliferation	Cell Motility	Lamellipodia Formation
ACTC1-KD	↓	↑	↑	↓	↓
ACTA2-KD	↑	↓	↓	↓	↓
ACTC1/ACTA2 double KD	↓	↓	↓	↓	↓

## Data Availability

All of the data analyzed in this study are available on reasonable request from the corresponding author.

## Supplementary Materials

The following supporting information can be downloaded at https://www.mdpi.com/article/10.3390/brainsci13101477/s1: Figure S1: Relationship between distant disease at first presentation and ACTA2 expression level; Figure S2: ACTA2 gene expression level by grade in WHO grade 2–4 clinical gliomas in the TCGA cohort; Figure S3: The Relationship between ACTA2 gene expression and survival prognosis of gliomas in the TCGA.
