# Supplementary material for: Actin Alpha 2, Smooth Muscle (ACTA2) Is Involved in the Migratory Potential of Malignant Gliomas, and Its Increased Expression at Recurrence Is a Significant Adverse Prognostic Factor
Source: Brain Sci. 2023 Oct 19;13(10):1477. doi: 10.3390/brainsci13101477 (PMC10605410; doi:10.3390/brainsci13101477)
Supplement: Supplementary file 1 [file brainsci-13-01477-s001.zip › brainsci-2660733-supplementary.pdf]

Supplementary Materials

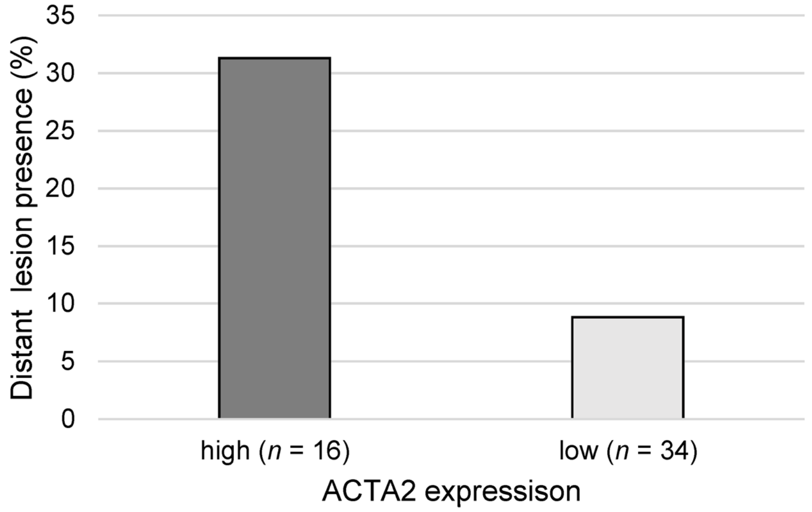

Figure S1. Relationship between distant disease at first presentation and ACTA2 expression level. We divided patients with primary malignant glioma (n = 50) into two groups: the top third with high ACTA2 gene expression (n = 16) and the remaining two-thirds of cases (n = 34) and compared the proportion of cases with distant lesions that were not contiguous with the main tumor mass using Gd contrast-enhanced MRI images of the brain at initial onset. The ACTA2 high expression group (n = 16) was significantly more likely to have distant lesions (31.3%) than the ACTA2 low expressing group (n = 34, 8.8%) (p = 0.044). Statistical analysis was performed using Pearson's chi- square test.

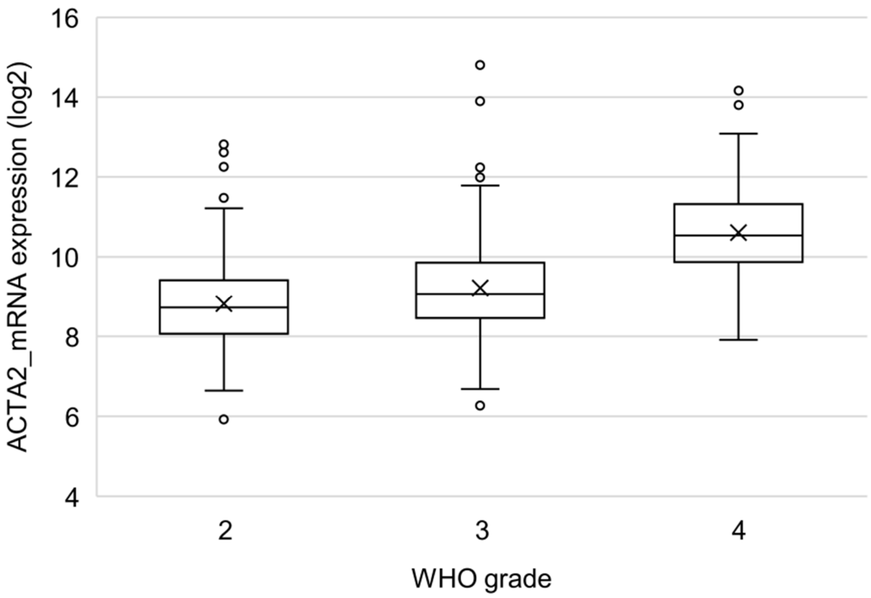

Figure S2. ACTA2 gene expression level by grade in WHO grade 2-4 clinical gliomas (n = 667) in the TCGA cohort. ACTA2 expression was significantly higher in grade 4 gliomas (n = 153) compared to grade 2 (n = 226) and grade 3 gliomas (n = 244) ($p < 0.001$). Analysis of variance was performed to obtain the p-value.

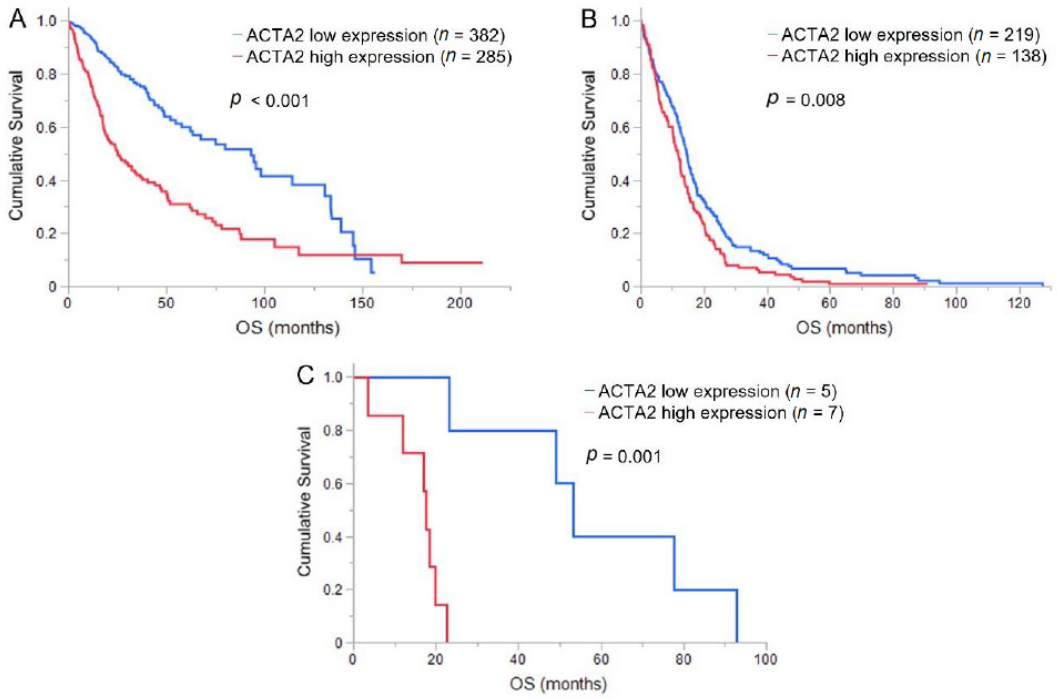

Figure S3. The Relationship between ACTA2 gene expression and survival prognosis of gliomas in the TCGA. **A**: Among WHO grade 2-4 glioma patients (n = 667), those in the high ACTA2 expression group (n = 285) showed significantly shorter overall survival (OS) compared to those in the low ACTA2 expression group (n = 382) ($p < 0.001$). **B**: In primary glioblastoma (GBM) cases with IDH-wild type (n = 357), the high ACTA2-expressing group (n = 138) exhibited significantly shorter OS in comparison to the low ACTA2-expressing group (n = 219) ($p = 0.008$). **C**: Among 12 patients with recurrent glioblastoma of IDH-wild type, the high ACTA2 expression group (n = 7) had notably shorter OS than the low ACTA2 expression group (n = 5) ($p = 0.001$). We used the maximum selection rank statistic to determine these groupings' optimal cutoff value for ACTA2 expression. Statistical significance was determined using the log-rank test.
